# Supplementary figures and images for: Dieting alleviates hyperuricemia and organ injuries in uricase-deficient rats via down-regulating cell cycle pathway
Source: PeerJ. 2023 Sep 8;11:e15999. doi: 10.7717/peerj.15999 (PMC10494837; doi:10.7717/peerj.15999)

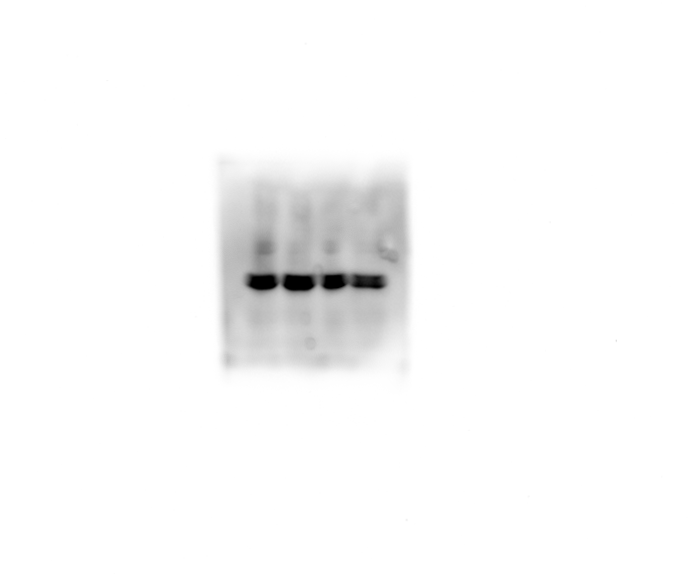

Supplement: Supplemental Information 31 [file peerj-11-15999-s031.tif]

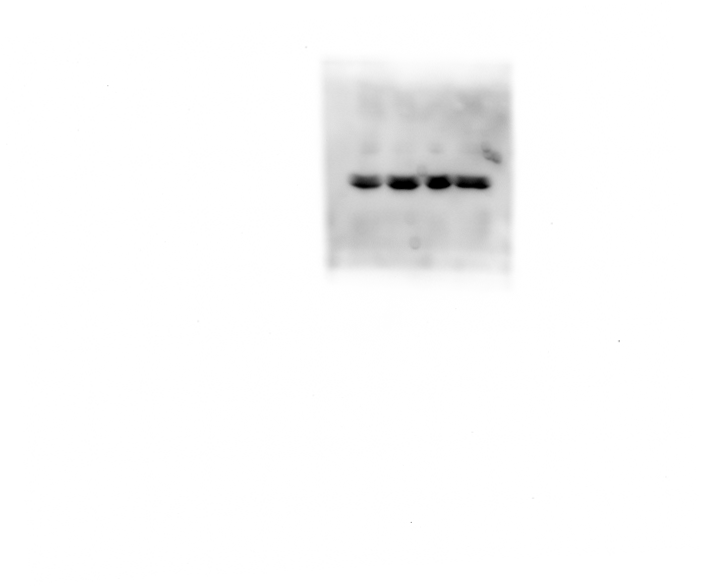

Supplement: Supplemental Information 32 [file peerj-11-15999-s032.tif]

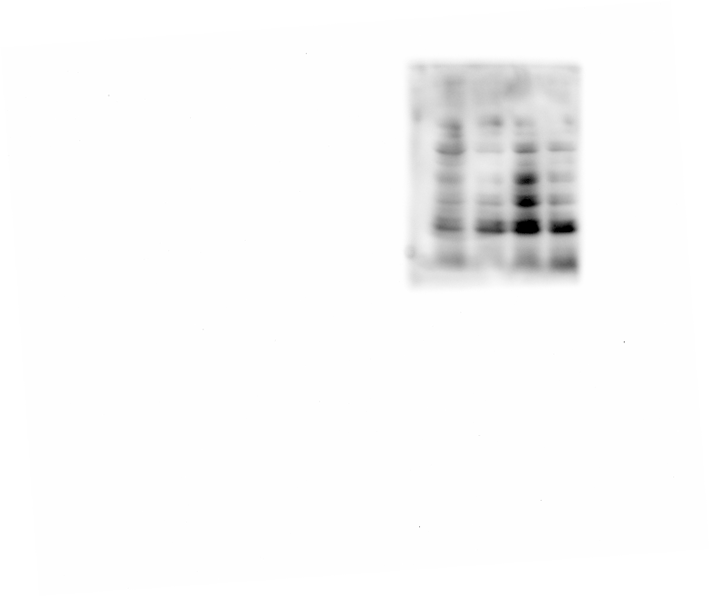

Supplement: Supplemental Information 33 [file peerj-11-15999-s033.tif]

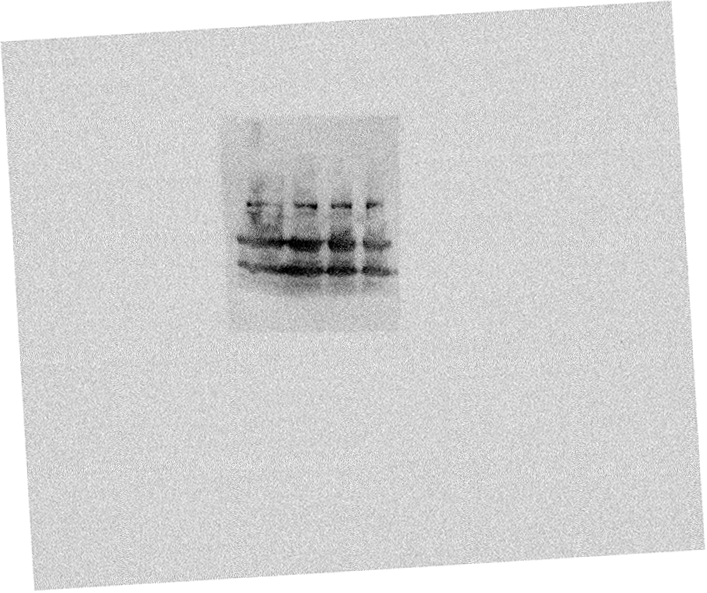

Supplement: Supplemental Information 34 [file peerj-11-15999-s034.tif]
